# Structural Variations in LysM Domains of LysM-RLK *Ps*K1 May Result in a Different Effect on Pea–Rhizobial Symbiosis Development

**DOI:** 10.3390/ijms20071624

**Published:** 2019-04-01

**Authors:** Anna N. Kirienko, Nadezhda A. Vishnevskaya, Anna B. Kitaeva, Oksana Yu. Shtark, Polina Yu. Kozyulina, Richard Thompson, Marion Dalmais, Abdelhafid Bendahmane, Igor A. Tikhonovich, Elena A. Dolgikh

**Affiliations:** 1 All-Russia Research Institute for Agricultural Microbiology, Podbelsky chausse 3, Pushkin, 196608 St. Petersburg, Russia; kirienkoann@yandex.ru (A.N.K.); navishnevskaya@rambler.ru (N.A.V.); anykitaeva@gmail.com (A.B.K); oshtark@yandex.ru (O.Y.S.); polykoz@gmail.com (P.Y.K.); arriam2008@yandex.ru (I.A.T.); 2 Agroécologie, AgroSup Dijon, INRA, Univ. Bourgogne Franche-Comté, 21000 Dijon, France; richard.thompson@inra.fr; 3 IPS2, UMR9213/UMR1403, CNRS, INRA, UPSud, UPD, SPS, 91405 Orsay, France; marion.dalmais@inra.fr (M.D.); abdelhafid.bendahmane@inra.fr (A.B.)

**Keywords:** legume–*Rhizobium* symbiosis, LysM receptor-like kinases, Nod factor perception, bacterial mutants, arbuscular mycorrhizal fungi, *Fusarium culmorum* fungi, pea *Pisum sativum* L.

## Abstract

Lysin-motif receptor-like kinase *Ps*K1 is involved in symbiosis initiation and the maintenance of infection thread (IT) growth and bacterial release in pea. We verified *Ps*K1 specificity in relation to the Nod factor structure using *k1* and rhizobial mutants. Inoculation with *nodO* and *nodE nodO* mutants significantly reduced root hair deformations, curling, and the number of ITs in *k1-1* and *k1-2* mutants. These results indicated that *Ps*K1 function may depend on Nod factor structures. *Ps*K1 with replacement in kinase domain and *Ps*SYM10 co-production in *Nicotiana benthamiana* leaves did not induce a hypersensitive response (HR) because of the impossibility of signal transduction into the cell. Replacement of P169S in LysM3 domain of *Ps*K1 disturbed the extracellular domain (ECD) interaction with *Ps*SYM10′s ECD in Y2H system and reduced HR during the co-production of full-length *Ps*K1 and *Ps*SYM0 in *N. benthamiana*. Lastly, we explored the role of *Ps*K1 in symbiosis with arbuscular mycorrhizal (AM) fungi; no significant differences between wild-type plants and *k1* mutants were found, suggesting a specific role of *Ps*K1 in legume–rhizobial symbiosis. However, increased sensitivity to a highly aggressive *Fusarium culmorum* strain was found in *k1* mutants compared with the wild type, which requires the further study of the role of *Ps*K1 in immune response regulation.

## 1. Introduction

Lysin motif receptor-like kinases (LysM-RLKs) are specialized plant receptors involved in the recognition of compounds consisting of N-acetyl-D-glucosamine (GlcNAc) residues. In this regard, LysM-RLKs play an important role in the recognition of compounds stimulating immune reactions in plants (chitin, peptidoglycan, and their derivatives) as well as other molecules triggering symbiosis development with nitrogen-fixing bacteria and arbuscular mycorrhizal (AM) fungi (lipo-chitooligosaccharide (LCO) signals Nod factors, Myc factors and short chitooligosaccharides (CO4-5).

Over the last decade, a number of such receptors, which are predominantly involved in the perception of chitin, peptidoglycan, and their derivatives, have been reported in various plant species. In turn, this has allowed for the elucidation of the possible mechanisms of ligand binding and receptor operation. An analysis of the LysM-RLKs *At*CERK1 and *Os*CERK1 in model plants *Arabidopsis thaliana* and rice *Oryza sativa* showed that the formation of multicomponent complexes with other LysM-containing proteins is a likely basis of their operation. The complexes between *At*CERK1 and LysM-RLKs with a non-active kinase domain, *At*LYK5 and *At*LYK4, *Os*CERK1 and LysM-receptor-like protein (LysM-RLP) *Os*CEBiP, are involved in the recognition of chitin and its derivatives [1,2,3,4,5,6,7]. To recognize the peptidoglycan, trimeric complexes such as *At*CERK1 and LysM-RLPs *At*LYM1, *At*LYM3 as well as *Os*CERK1 and LysM-RLPs *Os*LYP4, *Os*LYP6 are formed [8,9].

An analysis of the crystal structures of *At*CERK1 and *Os*CEBiP with ligand (chitin and CO8) and molecular modeling revealed the existence of a certain site on the surface of the LysM domains, a shallow groove that interacts with the GlcNAc-containing backbone of these molecules [5]. A crystal structure of the *At*CERK1 ectodomain (ECD) showed the binding of three GlcNAc-residues in a shallow groove to one of the three LysM motifs, the LysM2 of *At*CERK1 [5]. The activation model suggests that CO8 acts as a bivalent ligand, inducing *At*CERK1/*At*CERK1 or *At*LYK5/*At*CERK1 dimerization and kinase activation [5,6]. In rice, one more activation model was proposed in which CO8 activated the dimerization of *Os*CEBiP molecules that proceeded by the addition of two *Os*CERK1 molecules in a common heterocomplex [10]. The crystallization of *Os*CEBiP with CO4 also showed the direct binding of three GlcNAc-residues in a shallow groove in the LysM2-motif of this LysM-RLK [11]. Recently, in legume plants, the closest homologues of CERK1, the chitin-binding LysM-RLKs *Lj*LYS6 from *Lotus japonicus, Mt*LYK9 from *Medicago truncatula* and *Ps*LYK9 from *Pisum sativum*, were identified [12,13]. Molecular modeling with individual LysM2 domains showed a potential chitin-binding site in a shallow groove [13].

A number of LysM-RLKs involved in LCO binding and symbiosis development are also believed to form receptor complexes for signal transduction [14]. Members of LysM-RLKs are subdivided into two subclasses: namely, LYK and LYR. In *Lotus japonicus*, two LysM-RLKs *Lj*NFR1 (LYK) carrying an active kinase domain and *Lj*NFR5 (LYR) with a non-functional kinase are both equally essential for symbiosis formation and can bind Nod factors [15,16]. To transmit a signal inside the cell, *Lj*NFR5 may be combined into a heterocomplex with *Lj*NFR1 [15,17]. In legumes such as *M. truncatula* and *P. sativum*, that form a non-determined type of nodules, genes homologous to *LjNFR5* (*MtNFP/PsSym10* and *MtLYR3*/*PsLYR3*) and *LjNFR1* (*MtLYK3/PsSym37* and *PsK1*) were found [15,18,19,20,21,22,23,24,25]. As shown by the analysis of mutants impaired in these genes or binding experiments, all of these are essential genes for symbiosis development. This finding suggests a more complicated mechanism for Nod factor perception, most likely involving several receptor complexes in perception and subsequent signal transduction [21,25,26,27,28,29]. Indeed, the possibility of dimerization between *Mt*NFP/*Mt*LYK3, *Mt*LYR3/*Mt*LYK3 and *Ps*SYM10/*Ps*K1 and *Ps*SYM10/*Ps*Sym37 was shown using FRET analysis and other approaches such as the co-expression of receptors in *Nicothiana benthamiana* leaves and a yeast two-hybrid system [25,30,31,32]. However, how the symbiotic LCO signals are perceived and transmitted via these complexes is not clear. Further, the functional role of LysM-RLK dimerization is far from clearly understood and is not connected with certain sites in proteins.

The molecular modeling of individual LysM domains of the LYR receptors such as *Mt*NFP, *Lj*NFR5 and *Mt*LYR3 showed the possibility of Nod factor binding in a standard shallow groove on their surface [23,24,33,34]. Based on the analysis of mutants, the functional importance of amino acid positions in individual LysM domains such as Leu118 in LysM2 of *Lj*NFR5 [34], Leu154 in LysM2 of *Mt*NFP [29] and Tyr228 in LysM3 of *Mt*LYR3 [24] was shown. All of these are located in the regions between the β layer and α helix in the LysM domain, comprising borders of a standard shallow groove [35].

However, the structure–function analysis of ligand recognition for LysM-RLKs from the LYK group involved in LCO perception was not performed. In pea, we recently showed an important role of the LysM-RLK *Ps*K1, which belongs to the LYK group [25] in symbiosis development. A set of mutants impaired at the *Psk1* gene was obtained (Table 1) using the TILLING approach [25,36]. Based on the phenotypical characterization of *k1* mutant lines, it was revealed that *Ps*K1 is involved in the initiation of the symbiosis in a complex with *Ps*SYM10 and may also control the development of ITs in cortex and bacterial release. These mutants provide a unique opportunity to study the effect of individual amino acids on ligand binding and the receptor’s ability to form a complex with *Ps*SYM10. Recently, the modeling of Nod factor binding with homologs of pea *Ps*K1 and *Ps*SYM10 in *Vicia sativa* (*Vs*K1 and *Vs*NFR5) showed an importance of receptor heterodimerization for ligand binding that was consistent with our data for peas [35]. To estimate the specificity of Nod factor recognition by the *Ps*K1, we used a set of *Rhizobium leguminosarum* bv. *viciae nodE, nodO* and *nodE nodO* mutants producing Nod factors. 

A special role of *Ps*K1 may be connected with the regulation of rhizobial release into the host cell. However, it is still unknown whether this function can be extended to the interaction with other symbiotic microorganisms, such as AM fungi. It is also important to estimate the role of *Ps*K1 in the regulation of the resistance to invasion by phytopathogens. The homologues of *Ps*K1, *Lj*NFR1 and *Mt*LYK3 were shown to be important not only for rhizobial symbiosis, but also for mycorrhizal infection [37,38]. LysM-RLK *Mt*NFP plays an important role in the development of early reactions during AM symbiosis [39,40], but also regulates the plant resistance to phytopathogens [41]. To verify the LysM-RLK function, we have also estimated a possible role of *Ps*K1 in symbiosis with AM fungi and resistance to phytopathogens. 

## 2. Results

### 2.1. Effect of R. Leguminosarum bv. Viciae Strain A34 and Its Derivatives nodE (A42), nodO (A67) and nodE nodO (A91) Rhizobial Strains on Pea Mutants Impaired in the k1 Gene

Previously, *k1-1* and *k1-2* mutants with loss-of-function Nod^-^ phenotypes and the *k1-3* mutant with delayed infection development were identified in pea [25]. We used these mutant lines to verify the specificity of LysM-RLK *Ps*K1 in relation to the Nod factor structure. In contrast to the *k1-1* mutant line, which was almost completely blocked in response to inoculation, the *k1-2* mutant was impaired in the maintenance of IT growth in cortex and rhizobia penetration, while the *k1-3* mutant was found to have a delay in infection at 14 dai, which was restored to 21 dai. We compared the responses of wild-type pea plants cv. Cameor and *k1-1, k1-2 and k1-3* mutant plants to inoculation with rhizobial mutant strains *nodE* (A42), *nodO* (A67) and *nodE nodO* (A91), which are derivatives of parental strain A34 [42].

**Figure 1:**
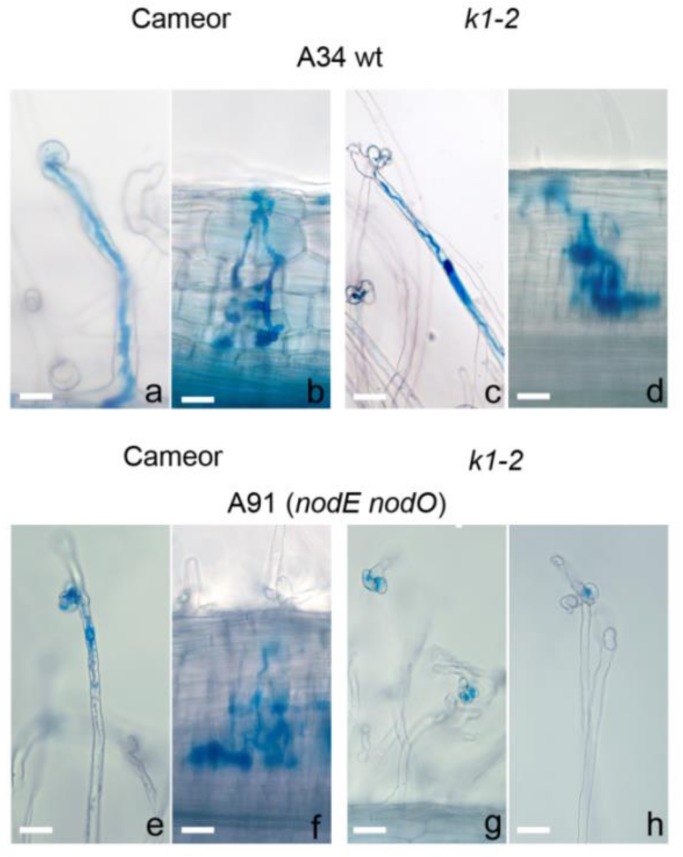
(**a**–**d**) Light micrograph of wild-type and *k1-2* mutant roots inoculated with *R. leguminosarum strain* A34 at 14 dai. (**a**) Root hair curling and microcolony in wild-type (WT) plants. (**b**) Normal infection thread (IT) growth and primordium development in WT plants. (**c**) Root-hair curling containing a microcolony and the development of sac-like IT in root hair in *k1-2* line roots. (**d**) Transcellular growth of sac-like IT with no bacteria release in *k1-2* line roots. (**e**–**h**) Light micrograph of wild-type and *k1-2* mutant roots inoculated with *R. leguminosarum* strain A91 (*nodE nodO* mutant) at 14 dai. (**e**) IT blocked in root hair of WT roots. (**f**) IT development and primordium formation in WT roots. (**g**,**h**) Only root hair curlings and microcolonies in *k1-2* line roots were observed. Bars: (**a**,**e**) = 50 μm; (**b**–**d**) = 40 μm; (**f**) = 50 μm; (**g**,**h**) = 30 μm.

**Figure 2:**
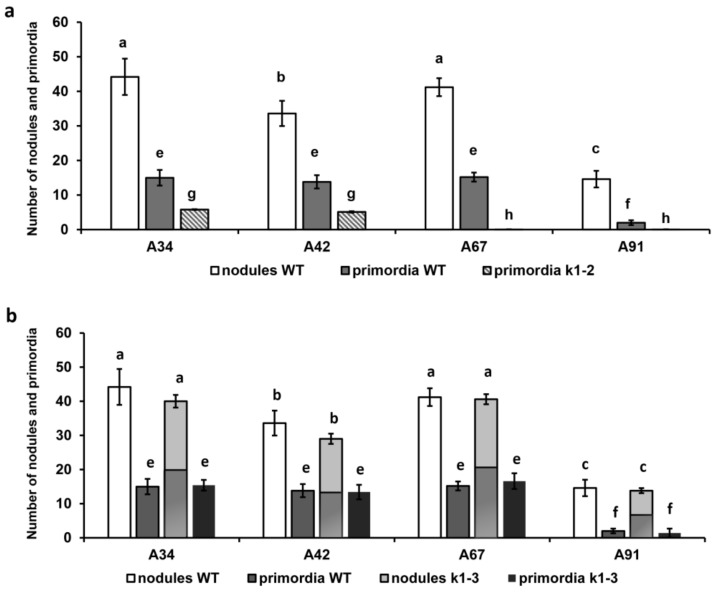
(**a**) Number of nodules and primordia on wild-type Cameor and *k1-2* mutant line roots during inoculation with set of *R. leguminosarum* strains per plant. (**b**) Number of nodules and primordium on wild-type Cameor and *k1-3* mutant line roots during inoculation with set of *R. leguminosarum* strains per plant. A part of nodules in *k1-3* mutant remained uninfected at 14 dai (53.7% for A42, 49.8% for A67 and 52.2% for A91). The *k1-1* mutant did not form the nodules and primordia and was not included in the analysis. Values are means ± SEM of three biological repeats (10 plants per variant in each repeat). Values are means ± SEM of three biological repeats (10 plants per variant in each repeat). Two-way ANOVA showed significant differences in the number of nodules and primordia, depending on the bacterial strain (F(df = 3)=376.08, *p* < 0.001 and F(df = 3) =536.44, *p* < 0.001) and depending on the type of plant (F(df = 2) = 19240, *p* < 0.001 and F(df = 2) = 597.01, *p* < 0.001). The interaction of the two factors also turned out to be significant (F(df = 6) = 94.94, *p* < 0.001) and F(df = 6) = 76.95, *p* < 0.001). Values with different letters are significantly different (*p* < 0.001) as analyzed by two-way ANOVA and the Tukey test (Table S3 for nodules and Table S4 for primordium).

The inoculation of wild-type plants with a *nodE* mutant strain producing C_18:1_ instead of C_18:4_ acyl chain increased the number of deformed and curled root hairs by 1.5-fold and slightly reduced the number of nodules and primordia compared with A34 (Table 2, Figure 1 and Figure 2). The *nodO* rhizobial gene encodes a secreted protein containing repeated calcium-binding domains in the N-terminal region (Sutton et al. 1993), but the *nodO* mutant had no significant effect on the nodulation of the wild type. However, inoculation with the *nodE nodO* double mutant significantly increased the number of aborted ITs in root hair cells that resulted in a reduced amount of the nodule/primordia number (Table 2). These results were in accordance with previous reports [27,43].

As was shown previously with other wild-type rhizobial strains, the inoculation of *k1-1* mutant with parental strain A34 almost completely blocked the responses to inoculation, only demonstrating very rare short aborted infection threads in small root hairs [25]. The inoculation of *k1-1* plants with the *nodE* mutant caused a slight increase of the number of such aborted infection threads. At the same time, inoculation with the *nodO* mutant entirely prevented any responses to inoculation, similar to the *nodE nodO* double mutant (Table 2).

As was consistent with our previous data, a decreased number of root hair deformations and curling and only rare cortical cell divisions were observed in the *k1-2* mutant inoculated with parental strain A34 (Table 2, Figure 2) [25]. The number of ITs was higher in this case, compared with wild-type plants cv. Cameor, but most of them were blocked in the outer cortex and no infected primordia were found (due to defect in bacterial release, as shown previously) [25]. Although the *nodE* mutant induced more deformations and curling compared with the A34 strain, the total number of deformed root hairs remained two-fold lower than in cv. Cameor. No significant influence on the IT number was observed in this case. Surprisingly, inoculation with another *nodO* mutant strain showed a significantly decreased amount of deformation and curling, as well as ITs (Table 2). Most ITs were blocked in the outer cortical layer. A more pronounced effect of the *nodE nodO* double mutant strain was shown in our experiments. Significantly fewer deformed and curled root hairs, and complete blocking of IT development and primordia formation, were recorded in this case (Table 2, Figure 1g, h and Figure 2).

The *k1-3* mutant displayed a “weak” phenotype which was basically similar to wild-type cv. Cameor [25]. The inoculation of *k1-3* mutant plants with parental A34 strain induced the formation of a nodule number comparable with wild-type plants; however, as shown previously [25], only a portion of them (about 54%) were infected at 14 dai (Figure 2b). An increased number of ITs was found in *k1-3* inoculated with A34, *nodE* and *nodO* strains compared with wild-type cv. Cameor, probably due to the compensatory effect. The inoculation of *k1-3* with *nodE* mutant strain increased the number of deformed and curled root hairs, as was shown in wild-type cv. Cameor for this rhizobial strain (Table 2). No significant differences in the number of deformations and curling as well as ITs were found during the inoculation of the *k1-3* mutant with a *nodO* mutant strain compared with the A34 strain. The number of nodules during *nodE* and *nodO* inoculation was also similar to the parental strain, while the tendency to have only a part of them infected remained. The inoculation of *k1-3* with the *nodE nodO* double mutant significantly increased the number of aborted ITs in root hair cells, resulting in a reduced amount of the nodule/primordia number, similar to the influence of this strain on cv. Cameor (Table 2). 

### 2.2. Analysis of Dimerization between PsSYM10 and PsK1 Carrying Replacements in LysM1, LysM3 and Kinase Domains in Various Systems

Previously, we showed the possibility of *Ps*SYM10 and *Ps*K1 interaction in two different heterologous systems [25]. The co-expression of full-length *Ps*SYM10 and *Ps*K1 in *N. benthamiana* leaves resulted in the development of a hypersensitive response (HR), thus providing evidence for the cooperative effect of proteins in this system. The interaction of extracellular domains (ECDs) of *Ps*K1 and *Ps*SYM10 was also demonstrated using the yeast two-hybrid (Y2H) assay, which showed that *Ps*SYM10 and *Ps*K1 have the potential to form hetero-oligomers between their ECDs (Kirienko et al. 2018). Here, we used the same two approaches to analyze the influence of amino acid replacements in various domains of *Ps*K1 on its interaction with *Ps*SYM10.

#### 2.2.1. Transient Co-Production of Pea LysM-RLKs in N. Benthamiana Leaves

**Figure 3:**
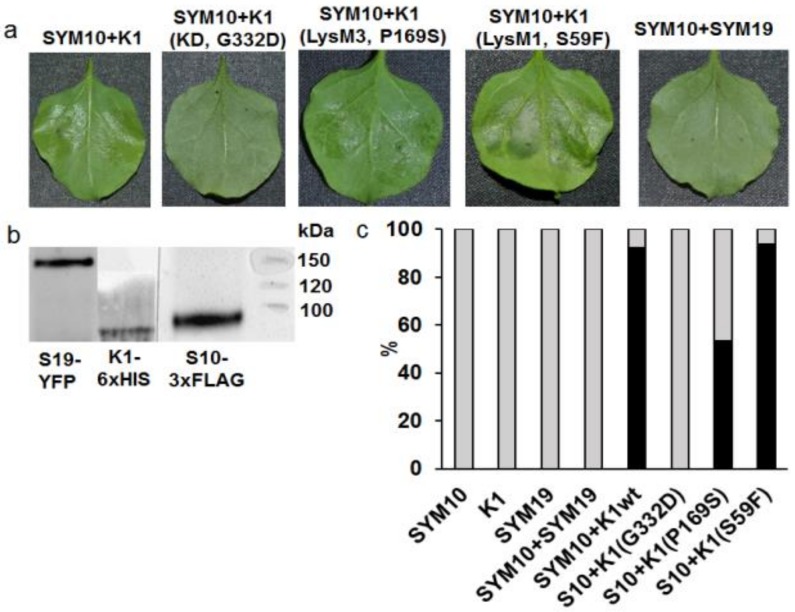
Analysis of hypersensitivity reaction (HR) development at the transient co-production of LysM-RLKs *Ps*SYM10 and *Ps*K1 (corresponding to proteins in wild-type (wt) and *k1-1*, *k1-2* and *k1-3* mutant lines) in *N. benthamiana* leaves. (**a**) The development of HR at the co-production of SYM10 + K1 (wt), SYM10 + K1 *(k1-2)* and SYM10 + K1 *(k1-3)*. No HR was revealed during SYM10 + K1 *(k1-1)* co-production (3 days after transformation). No HR was found at the co-production of SYM10 + SYM19 (negative control). (**b**) Western blot analysis of K1, SYM10, and SYM19 in leaves of *N. benthamiana* with anti-GFP, anti-6xHIS and anti-3xFLAG antibodies. The solubilized proteins were separated by gel electrophoresis in the presence of SDS and visualized using HRP-coupled antibodies together with Clarity Max Western ECL Substrate (Bio Rad, USA) staining solutions. (**c**) HR development is shown as the percentage (%) of leaves with HR (black) and healthy (gray) leaves after infiltration with bacteria harboring the corresponding constructs. Three independent experiments were performed (10–12 plants for variant in each experiment).

*Ps*SYM10-3xFLAG and *Ps*K1-6xHIS fusions were co-produced in *Nicotiana* leaves and induced a cell death response (a hypersensitive response (HR) of about 90%) 48–72 h after infiltration followed by cell disruption in 5–6 days, which was consistent with our previous data (Figure 3). In contrast, mono-transformation with wild-type full-length *Ps*SYM10-3xFLAG and *Ps*K1-6xHIS did not result in HR development in *N. benthamiana* leaves. To confirm the presence of synthesized proteins in the cells of *N. benthamiana*, Western blot analysis with antibodies against 6xHIS or FLAG was carried out (Figure 3b). The proteins with their expected molecular weights were detected on the blots.

All mutant variants were prepared as fusions on the C terminus with 6xHIS. It was shown that at the co-production of *Ps*SYM10 and *Ps*K1 with the replacement G332→D in the kinase domain (corresponding to protein in *k1-1* mutant line), HR development was not observed (Figure 3a). This strongly suggests that the kinase domain of *Ps*K1 is involved in the activation of the signal transduction pathway in the cells. At the same time, during the co-production of *Ps*SYM10 and *Ps*K1 with replacement in LysM3 domain P169→S (corresponding to protein in *k1-2* mutant line), we observed a decrease in HR development by up to 53%. This underlines the importance of P169 in the LysM3 domain of *Ps*K1 for dimerization with another LysM-RLK, and subsequent signal transduction.

Finally, the co-production of *Ps*SYM10 and *Ps*K1 with replacement in the LysM1 domain S69→F (corresponding to protein in *k1-3* mutant line) did not lead to a significant change in HR development, as it was comparable with the reaction between wild-type *Ps*SYM10 and *Ps*K1 (about 93%) (Figure 3c). This may indicate that the replacement in the LysM1 domain of the *Ps*K1 is not critical for complex formation and signal transduction.

Since all interactions between *Ps*SYM10 and mutant variants of *Ps*K1 developed in cells without ligand mediation, the Nod factor, this demonstrates the importance of mutual protein complementarity for effective signal transmission into the cell. *Ps*SYM19 was used as a negative control (orthologue of *Lj*SYMRK/ *Mt*DMI2) [44,45]. The co-production of *Ps*SYM10 and *Ps*SYM19 did not lead to HR development, which was consistent with our previous results (Figure 3). 

#### 2.2.2. Yeast Two-Hybrid Assay (GAL4 Transcription Factor-Based Assay)

**Figure 4:**
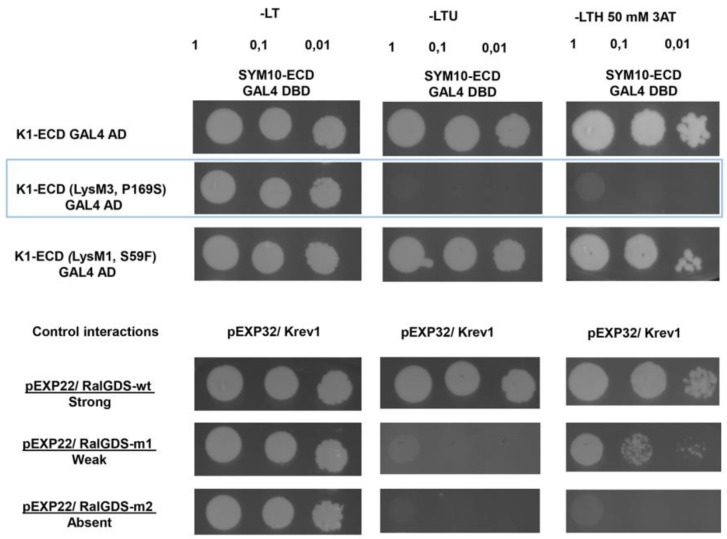
Yeast two-hybrid assays between SYM10-ECD and K1-ECD, K1-ECD (with P169S replacement in LysM3,), K1-ECD (with S59F replacement in LysM1). A yeast two-hybrid assay was performed using the extracellular domains of *Ps*SYM10, *Ps*K1 (corresponding to proteins in wild-type and *k1-2* and *k1-3* mutant lines). Serial dilutions OD_600_ = 1, 0.1 and 0.01 were used. Yeast growth on a selective medium lacking leucine and tryptophan (SD-LT) indicated the presence of both, bait and prey constructs. Interaction was tested on a medium additionally lacking uracil (SD-LTU) or supplemented with 3-amino-1,2,4-triazole (3AT) (SD-LTH + 50 mM 3AT). As controls, a few pairs of vectors (pEXP32/Krev1 and pEXP22/RalGDS-wild type, pEXP22/RalGDS-m1, and pEXP22/RalGDS-m2) suggested by the manufacturer were used for strong, weak, and not detectable interactions (Thermo Fisher Scientific).

Our previous experiments showed that ECDs of *Ps*SYM10 and *Ps*K1 may interact in the yeast two-hybrid system, suggesting the possibility of dimerization between two proteins. Since only two replacements in the LysM1 domain (corresponding to protein in *k1-3* mutant) and LysM3 domain (corresponding to protein in *k1-2* mutant) of *Ps*K1-ECD were previously found, the analysis was done for these two variants. We found that replacement in the LysM1 domain of the *Ps*K1-ECD did not affect complex formation with *Ps*SYM10-ECD. In contrast, replacement in the LysM3 domain of the *Ps*K1-ECD prevented complex formation in the yeast two-hybrid system (Figure 4). It can be concluded that a mutation leading to amino acid replacement P169→S in the *Ps*K1 ECD prevents complex formation with *Ps*SYM10, which is probably critical for effective ligand recognition and subsequent signal transduction. 

### 2.3. Effect of LysM-RLK PsK1 on Symbiosis Development with AM Fungus

**Figure 5:**
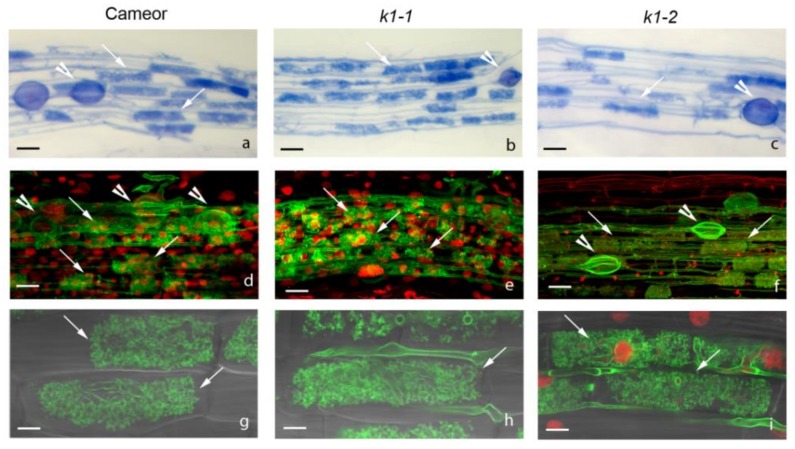
(**a**–**c**) Arbuscular mycorrhizal fungus colonizes efficiently wild type, *k1-1* and *k1-2* in a nurse plant system. Normal arbuscular mycorrhizal (AM) fungus colonization in wild type (**a**), *k1-1* (**b**) and *k1-2* lines (**c**). Arbuscules, vesicles are formed. (**d**–**i**) Confocal microscope images of wild-type (**d**) and mutant *k1-1* (**e**) and *k1-2* (**f**) *P. sativum* L. line roots colonized with *R. irregularis*. The roots at 3 dpi were stained with Alexa Fluor 488 WGA and propidium iodide. White arrows indicate cells with arbuscules, and white arrowheads point at vesicles. No different in arbuscule formation inside root cells of *k1-1* (**h**), *k1-2* (**i**) were identified in comparison with the wild type (**g**). Scale bars: (**a**–**d**,**f**) = 50 μm, (**e**) = 40 μm, (**g**,**h**) =10 μm, (**i**) = 15 μm.

**Figure 6:**
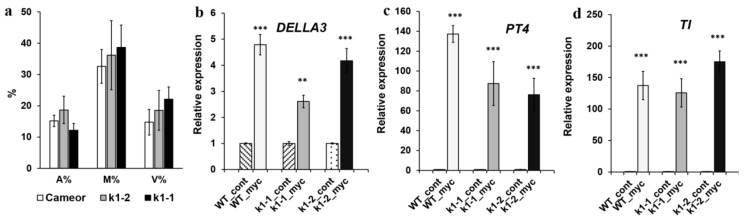
(**a**) Wild-type, *k1-1* and *k1-2* lines show a similar degree of fungal colonization (M%), and arbuscule (A%) and vesicle (V%) formation. (**b**–**d**) Transcript levels of marker genes *DELLA3* (a transcription factor involved in the development of symbiosis with AM fungus) (**b**), *PT4* (mycorrhiza-inducible inorganic phosphate transporter) (**c**) and TI (trypsin inhibitor genes, marker of arbuscules development) (**d**). mRNA levels were normalized against *Ubiquitin* and *Actin*, and values were calculated as ratios relative to non-inoculated root expression levels. The data of three independent biological experiments were analyzed. Asterisks indicate significant differences compared to noninoculated roots: *** *p* < 0.001, ** *p* < 0.01.

In order to assess the possibility of *Ps*K1 participation in symbiosis development with AM fungi, we infected two pea mutant lines, *k1-1* and *k1-2,* with *R. irregularis* fungus using a nurse plant system. Signs of symbiosis development were monitored three weeks after infection. Analysis showed that in a nurse plant system, *k1-1* and *k1-2* mutant lines were able to enter symbiosis with AM fungus and did not show significant differences in colonization compared with wild-type plants (Figure 5). There were no essential differences in the number of arbuscules and vesicles between mutant and wild-type plants (Figure 5). In addition, qRT-PCR did not reveal any differences in the expression level of markers typical for symbiosis development with AM fungi. Among these markers, the *PT4* gene encoding mycorrhiza-inducible inorganic phosphate transporter, the TI encoding inhibitor of trypsin, as well as the *DELLA3* gene encoding regulator of gibberellin signaling were included (Figure 6). In this system, we did not find any evidence for *Ps*K1 participation in the development of symbiosis with AM fungus. However, the possibility of identifying some differences between mutant and wild-type plants in another experimental system (for example, during inoculation with a lower amount of AM fungus spores) could not be completely excluded. 

### 2.4. LysM-RLK PsK1 May Be Involved in Pea Resistance to Fungal Pathogen Fusarium Culmorum 334

**Figure 7:**
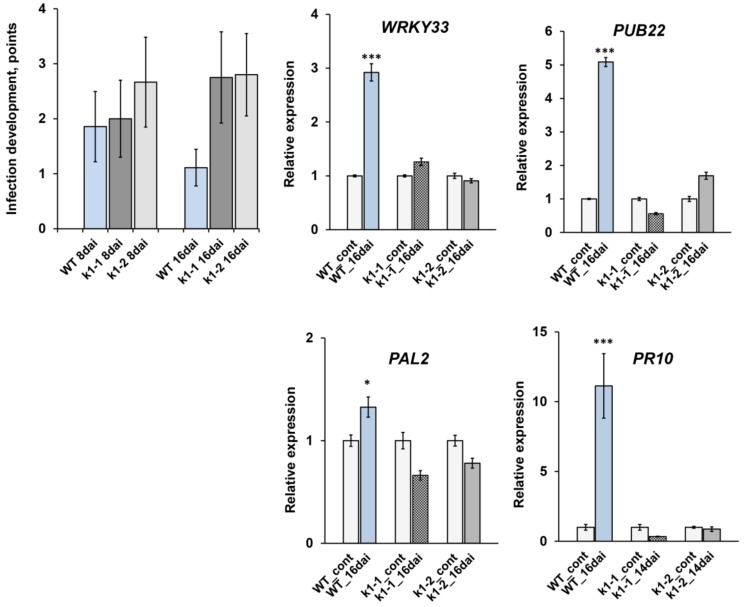
*k1-1* and *k1-2* possess reduced resistance to fungal pathogen *Fusarium culmorum* compared to wild-type plants cv. Cameor. The assessment of the level of the development of pea root rot in wild-type plants and mutant lines *k1-1* and *k1-2* at infection with *F. culmorum* strain 334 on the 8th and 16th day. Transcript levels of defense-response genes *WRKY33*, *PUB22, PAL2* and *PR10* at 16 dai in wild-type plants and *k1-1* and *k1-2* lines. mRNA levels were normalized against *Ubiquitin* and *Actin*, and values were calculated as ratios relative to non-inoculated root expression levels. The data of three independent biological experiments were analyzed. Asterisks indicate significant differences compared to untreated roots: *** *p* < 0.001, * *p* < 0.05.

To estimate a possible involvement of *Ps*K1 in the control of pea resistance to fungal pathogens, the *k1-1* and *k1-2* pea mutant lines were infected with pathogenic fungus *Fusarium culmorum* (Wm. G. Sm.) Sacc. strain 334 (a highly aggressive strain for pea). The symptoms of disease development were monitored at days 8 and 16 after infection using a five-point scale and compared to wild-type plants (Table S5). The effect of *F. culmorum* infection on root and shoot growth and development was also estimated. On day 8 after infection, in *k1-1* and *k1-2* mutant lines, the suppression of plant growth and development became visible, and signs of severe root rot were evident compared to the wild type. On day 16 after infection, we observed symptoms of the inhibition of plant growth and development in both mutant lines (shortening the length of stems and roots, Figure S1), and even the death of some of them. The infection also developed in wild-type plants, but this was not as evident as in plants of the *k1-1* and *k1-2* lines. Consistent with the visible symptoms, qRT-PCR analysis showed only the weak activation of defense-response genes *WRKY33*, *PUB22, PAL2* and *PR10* in plants of *k1-1* and *k1-2* mutant lines on day 16 after infection, in response to treatment with *F. culmorum*, compared with wild-type plants (Figure 7). The significant reduction of defense-response genes in *k1-1* and *k1-2* mutant plants, as well as the more severe symptoms of infection, may indicate the involvement of LysM-RLK K1 in the regulation of the development of immune reactions during pea invasion by phytopathogens. However, since there is no strict evidence to confirm that these effects of the pathogen are not due to a separate mutation causing increased plant sensitivity to invasion, additional studies are required to confirm the role of LysM-RLK K1 in such regulation.

## 3. Discussion

Little is known about the specificity of various LysM-RLKs from the LYK group in relation to Nod factor structure. Previously, a critical role in the control of bacterial infection dependent on Nod factor-acyl chain structure was shown only for LysM-RLK *Mt*LYK3 [19,21]. Moreover, the *PsSym37* gene, the putative *P. sativum* ortholog of *MtLYK3*, may be involved in the control of symbiosis development in a Nod factor structure-dependent manner as well [22,46]. Therefore, the question about the specificity of the LysM-RLK *Ps*K1 in symbiosis remains open.

Here, we conducted experiments with rhizobial mutants producing Nod factors with the C_18:1_ acyl chain instead of C_18:4_ and which were defective in NodO protein. In wild-type pea plants, the A42 (*nodE*) strain induced more intensive root hair deformations and IT growth. No essential effect on symbiosis development was shown with A67 (*nodO*) rhizobial mutant. However, the inoculation of wild-type pea plants with A91 (*nodE nodO*) double mutant significantly blocked IT growth and development, thus reducing the number of nodules. These results are in agreement with previous reports [27,47,48].

In contrast to wild-type plants, the analysis of pea *k1-1* and *k1-2* mutants revealed an essential effect of inoculation with the A67 (*nodO*) rhizobial mutant: the complete blocking of deformations and curling and aborted IT development in the *k1-1* line and a reduction of root hair deformations and curling, as well as the number of ITs in *k1-2* line. Analysis of *k1-1* and *k1-2* inoculation with A42 (*nodE*) mutant revealed no significant difference from parental A34 strain in the number of ITs. At the same time, inoculation with A91 (*nodE nodO*) double mutant completely blocked any reactions including root hair deformations and curling as well as IT growth in *k1-1* mutant plants. Inoculation of *k1-2* mutant with A91 (*nodE nodO*) completely blocked IT growth and development and primordium formation. 

Previous experiments on pea and vetch showed that the NodO protein stimulated the normal development of ITs by a strain unable to make host-specific substitutions to the Nod factors, an *R. leguminosarum* bv. *viciae nodFELMNTO* deletion mutant [27]. This suggested the participation of NodO rhizobial protein in a stimulation of the signal transduction pathway regulating IT development that probably compensated for the loss of an appropriate Nod factor structure [49]. It also suggested that secreted NodO protein due to the presence of calcium-binding repeats and the capacity to form cation-selective channels in lipid bilayers may regulate ion exchange in plant cells [47]. 

**Figure 8:**
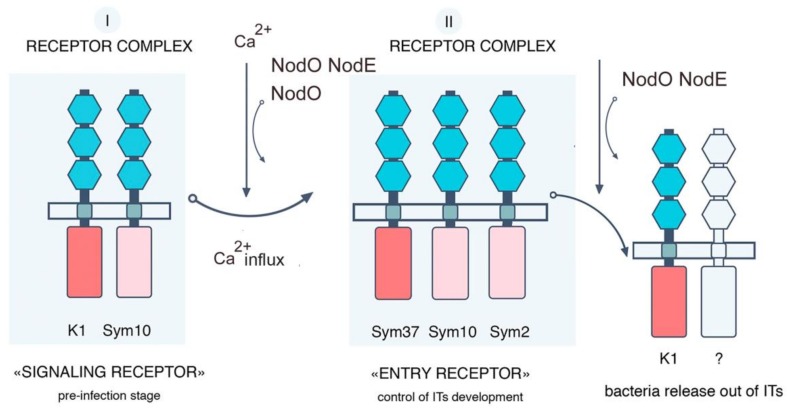
Model of receptor complexes organization in *P. sativum* L. and influence of NodO and NodE on the process controlled by receptor complexes [25].

In our experiments, a significant effect of A67(*nodO*) rhizobial mutant on two pea *k1-1* and *k1-2* mutants showed that LysM-RLK *Ps*K1 may be involved in the regulation of root hair deformations and curling in a NodO function-dependent manner (Figure 8). It seems like the functional NodO protein may partly compensate the signal transduction pathway in the *k1-2* mutant, while inoculation with A67 (*nodO*) mutant allowed this effect to show. Consistent with our previous assumption, the *Ps*K1/*Ps*SYM10 complex may regulate early symbiotic reaction including root hair deformations and curling (Figure 8) [25]. Previous studies have shown that Nod factors may induce two independent calcium responses in cells, such as an early calcium influx followed by calcium spiking in the perinuclear region and nucleus [50]. Calcium influx is one of the earliest responses to Nod factor perception in the roots; it precedes root hair deformations and is not caused by structural analogs of Nod factors, the COs or Nod factor, lacking some substitutions [50]. Since root hair deformations were almost completely blocked in *k1-1* mutant with strong phenotype [25], and the earliest detectable responses were not observed in the *sym10* mutant [15], it seems likely that both LysM-RLK SYM10 and K1 may regulate calcium influx in the plant cells. Since the NodO protein is capable of forming cation-selective channels in the membrane, we speculate that it may be involved in regulating the cellular ion exchange that controls root hair deformations and curling in pea roots.

We showed that the inoculation of *k1-1* and *k1-2* pea mutant with A91 (*nodE nodO*) double rhizobial mutant completely blocked IT growth and development and primordia formation. Consistent with earlier reports, these experiments confirmed that the recognition of the Nod factor by a specific acyl chain structure is important for the maintenance of IT growth in the epidermis and cortex, as well as primordia formation in pea [27,51]. These data suggest that the NodO protein can stimulate the signal transduction pathway, leading to IT growth maintenance. They also demonstrate that *Ps*K1 is involved in the regulation of these processes. This allowed us to offer an updated scheme of receptor complex organization in pea during the development of symbiosis with rhizobia (Figure 8).

One interpretation of these data is that the activation of early symbiotic reactions, including calcium influx by the *Ps*K1/ *Ps*SYM10 complex, may stimulate the assembly of another receptor complex regulating IT initiation and growth. Previously, we suggested that the receptor complex including *Ps*SYM10, *Ps*SYM37 and hypothetical *Ps*SYM2 may regulate IT initiation and growth in a Nod factor structure-dependent manner in pea [25]. Its participation in the stimulation of this complex may explain the effect of NodO protein and *Ps*K1 on the regulation of IT development. Since the inoculation of *k1-1* and *k1-2* mutants with A42 (*nodE*) strain producing Nod factors with a modified acyl chain showed a similar effect on IT initiation and growth as inoculation with A34 strain; seemingly, the early stages of symbiosis development—including root hair deformation and curling controlled by *Ps*K1—are not strictly dependent on Nod factor acyl-chain structure. 

An alternative explanation for the significant effect of the A91 (*nodE nodO*) strain on *k1-1* and *k1-2* mutants may be that *Ps*K1 is also involved in the maintenance of IT growth and bacterial release at later stages. As was suggested previously, *Ps*K1 may be involved in an additional receptor complex regulating IT distribution and rhizobia penetration [25]. In this case, the inoculation of two pea mutants with A91 (*nodE nodO*) double rhizobial mutant may have a more severe effect and completely block these processes. The development of these events may depend on the Nod factor acyl-chain structure, which implies the specificity of LysM-RLK *Ps*K1 to the ligand structure to form a complex with this additional receptor. 

The operation in the complex with another receptor may be important for ligand perception and subsequent signal transduction. Previously, we showed that the heterologous co-production of full-length *Ps*SYM10 and *Ps*K1 proteins in *N. benthamiana* leaves led to HR development, which allowed us to suggest a functional interaction of these two LysM-RLKs [25]. A similar effect was demonstrated for homologous *Lj*NFR5 and *Lj*NFR1, *Mt*NFP and *Mt*LYK3, which occurred in the system in the absence of a ligand [30,52]. Here, we showed that G332→D replacement in the nucleotide-binding glycine-rich loop of the kinase domain (encoded by *k1-1* gene) did not initiate HR development, most probably due to the impossibility of signal transduction into cells. Similarly, mutation in the *Mtlyk3-1* gene resulting in G334→E replacement in the nucleotide-binding loop prevented HR development in *N. benthamiana* leaves in *Medicago* [30].

Since the direct interaction of *Ps*K1-ECD and *Ps*SYM10-ECD in the Y2H system was also demonstrated, we speculated that a possible complementarity between their ECDs may be important for complex formation. Indeed, P169→S replacement in the LysM3 domain of *Ps*K1-ECD (encoded by *k1-2*) disturbed its interaction with *Ps*SYM10-ECD in the Y2H system significantly, while no essential effect was visible for S59→F replacement in the LysM1 domain of *Ps*K1-ECD (encoded by *k1-3*). This replacement in LysM3 domain also led to a decreased level of the HR during the co-production of the full-length *Ps*K1 (P169→S) and *Ps*SYM0 in *Nicotiana* leaves. Thus, we may conclude that this amino acid in the LysM3 motif is important for *Ps*K1 and *Ps*SYM10 complex formation and subsequent ligand perception.

Complex assembly before ligand perception was shown for some receptors. Thus, *Os*CERK1 and *Os*CEBIP were predicted to form a complex based on their co-immunoprecipitation in the absence of a ligand and interaction in the Y2H assay [10]. Similarly, *At*BAK1 and *At*BIR2 RLKs to flagellin from *A. thaliana* may be assembled in the complex, while ligand binding led to *At*BIR2 release and made *At*BAK1 available for another complex formation with *At*FLS2 [53]. If *Ps*K1 and *Ps*SYM10 may be combined into the complex before Nod factor perception, some amino acids should be important for such interaction and their replacement should lead to an inability to form a complex. Indeed, the data reported herein constitute the first evidence of this.

**Figure 9:**
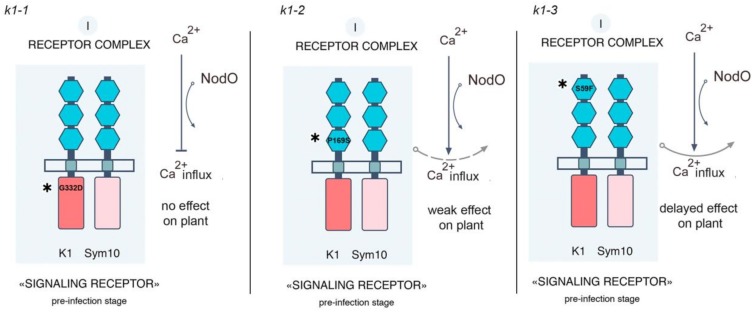
Scheme of signal transduction controlled by *Ps*K1/*Ps*SYM10 receptor complexes and the influence of NodO and mutations in *k1* gene on this process in *P. sativum* L. [25].

Indeed, the G332→D replacement in the kinase domain (encoded by *k1-1* gene) disrupted a signal transduction into the cell and led to the most significant disruption of symbiosis development (Figure 9). Since the inoculation of *k1-1* mutant with the A67 (*nodO*) strain completely blocked any responses from the plant side, it may be assumed that the calcium influx may be important for these initial stages. At the same time, the P169→S replacement in the LysM3 domain of *Ps*K1 (encoded by *k1-2*) disturbed the interaction with *Ps*SYM10, which attenuated the signal transduction and prevented the development of subsequent stages such as IT maintenance and bacterial release (Figure 9). An essential effect of the *nodO* mutant strain on the *k1-2* mutant line confirmed the importance of calcium in the activation of these stages. Finally, the S59→F replacement in the LysM1 domain of *Ps*K1 (encoded by *k1-3*) resulted only in the delayed infection of forming nodules, and no significant effect of *nodO* strain was found during the inoculation of this mutant (Figure 9). Since this replacement in the LysM1 domain did not influence the PsK1/PsSYM10 complex formation, it may suggest the participation of the LysM1 domain in Nod factor binding, which requires further verification. However, since the calcium influx seems to be active in this mutant, it did not have such a strong effect on symbiosis development.

Multiple studies have shown that some LysM-RLKs may be involved not only in legume–rhizobial symbiosis but may also be capable of regulating plant interactions with other micro-organisms. Thus, participation in the development of symbiosis with AM fungi was shown for close homologs of *Ps*K1 in other legume plants, for *Lj*NFR1 and *Mt*LYK3, as well as for LysM-RLK *Mt*NFP [37,39,40]. Here, we investigated a possible role of *Ps*K1 in the regulation of plant interaction with other micro-organisms. Using a nurse plant system, we did not find any significant differences in the development of symbiosis with AM fungi between wild-type plants and *k1* mutants. This may suggest the specific role of *Ps*K1 in legume–rhizobial symbiosis. However, based on data for other LysM-RLKs, the possibility of *Ps*K1 involvement in the regulation of pea interaction with AM fungi under different experimental conditions cannot be completely excluded.

Nevertheless, we have shown that *Ps*K1 may be involved in the control of the immune response during interaction with phytopathogenic fungi. Increased sensitivity to the highly aggressive *Fusarium culmorum* strain was shown for the *k1* pea mutant line. The inhibition of plant growth and development as well as the reduction in the transcription level of defense-related genes was observed in *k1* mutants compared to the wild type. Since the involvement of LysM-RLK *Ps*K1 in the regulation of bacterial penetration of plant cells was shown previously, it is important to investigate the mechanisms for the regulation of the immune response involving this receptor. Therefore, the role of LysM-RLK K1 in the regulation of immune reactions during pea invasion by phytopathogens should be investigated in additional experiments.

## 4. Materials and Methods

### 4.1. Bacterial Strains and Inoculation

*Rhizobium leguminosarum* biovar *viciae* wild-type strain RCAM 1026 (WDCM 966), the derivatives of the *R. leguminosarum bv. viciae* wild-type strain A34, carrying *nod* mutant genes on the pSYM pRL1JI plasmid, the mutant strains A42 (*nodE*::Tn5), A67 (*nodO93*::Tn3 HoHo1) and A91 (*nodE*::Tn5, *nodO*::Tn3) were used [42,43]. Strains A34 and A42 carry the plasmid pXLGD4 containing the constitutive *hemA* promoter fused to *lacZ* [54]. For plant inoculation, liquid cultures were grown in B^-^ medium [55], diluted up to optical density at 600 nm (OD_600_) 0.5 and applied to plant inoculation. 

### 4.2. Plant Material and Growth Conditions

The pea *Pisum sativum* L. seeds of wild-type cv. Cameor or corresponding *k1-1, k1-2, k1-3* mutant lines (Table 1) were sterilized with sulphuric acid for 5 min, washed 3 times with water, transferred on 1% water agar plates and germinated at room temperature in the dark. Four to five-day-old seedlings were transferred into pots with vermiculite saturated with Jensen medium [56], grown in a growth chamber at 21 °C in 16 h light/ 8 h dark cycles, at 60% humidity. 

*N. benthamiana* seeds were surface-sterilized with 10% hypochlorite for 10 min, washed 5 times with water and left for imbibition on a plate with sterile filter paper at 4 °C. All seeds were germinated in a big plastic box with soil for 7 days, and then transferred into individual pots with soil. Plants were grown at 23 °C with 16 h light/ 8 h dark cycles, at 60% humidity.

### 4.3. RNA Extraction and Quantitative Reverse Transcription PCR (qRT-PCR)

RNA extraction and qRT-PCR were performed as described previously [57]. Primer pairs for qRT-PCR are listed in Table S6. The qRT-PCR analysis was performed on a CFX-96 real-time PCR detection system with a C1000 thermal cycler (Bio-Rad Laboratories, Hercules, California., USA).

### 4.4. Generation of Constructs for Plant and Yeast Transformation

pBIN19 vectors for *N. benthamiana* transformation, carrying wild-type cv. Cameor *K1* full-length coding sequences and sequences with nucleotide replacements corresponding to mutations in *k1-1, k1-2* and *k1-3* lines were obtained. The *K1* full-length coding sequence without a stop codon was amplified using the cDNA of cv. Cameor as a matrix and inserted into pDONR221 vector (Thermo Fisher scientific, Waltham, MA, USA) with BP clonase (Thermo Fisher scientific, Waltham, MA, USA). Finally, it was cloned into the pBIN-GWY vector carrying 6xHIS using the LR clonase enzyme (Thermo Fisher scientific, Waltham, MA, USA). 

pDONR221 vectors with full-length *K1* genes with nucleotide replacements corresponding to mutations in *k1-1, k1-2* and *k1-3* lines were obtained using a site-directed mutagenesis kit (Thermo Fisher scientific, Waltham, MA, USA) according to the manufacturer’s protocol with corresponding primers (Table S6). At the next stage, they were subcloned into the pBIN-GWY using the LR clonase enzyme. All verified constructs were transferred into the *Agrobacterium tumefaciens* LBA 4404.

For the yeast two-hybrid system, the partial sequences of *K1* cv. Cameor encoding the extracellular domains (ECDs) and flanked with attb1/attb2 sequences were inserted in the pDONR221 vector with BP clonase and finally into the pDEST22 (PREY) or pDEST32 (BAIT) using the LR clonase enzyme (Thermo Fisher scientific, Waltham, MA, USA). pDONR221 vectors with *K1* partial sequences, carrying replacements corresponding to mutations in *k1-2* and *k1-3* lines, were obtained using a site-directed mutagenesis kit (Thermo Fisher scientific, Waltham, MA, USA) according to the manufacturer’s protocol with corresponding primers (Table S6) and then subcloned into destination vectors pDEST32 or pDEST22 using the LR clonase enzyme. All verified constructs were transferred into *Saccharomyces cerevisiae* MaV203 yeast strain (Thermo Fisher scientific, Waltham, MA, USA).

### 4.5. Transient Protein Expression in Nicotiana Benthamiana Leaves

The *A. tumefaciens* strain LBA 4404 was used for infiltration in *N. benthamiana* leaves. The bacterial culture was grown at 28 °C overnight, then centrifuged at 3000 g and resuspended in 10 mM MES-KOH, 10 mM MgCl_2_ and 0.5 mM acetosyringone upto culture density OD_600_ = 0.5. Bacterial cells were infiltrated into the leaves of 3-week-old *N. benthamiana*. Plants were analyzed 48–96 h after infiltration.

### 4.6. Western Blot Analysis of Leaf Tissues

The sample preparation, SDS-PAGE and blotting were performed as previously described [25]. Proteins were detected using monoclonal anti-FLAG M2 antibodies conjugated with horseradish peroxidase (HRP) (Sigma-Aldrich, St. Louis, MO, USA) at 1:5000 or anti-His antibodies (Sigma-Aldrich, St. Louis, MO, USA) at 1:2000. The anti-GFP antibodies (OriGene Technologies, Germany) were also used for detection at 1:1500, followed by secondary anti-rabbit antibodies linked with HRP (Sigma-Aldrich, St. Louis, MO, USA) at 1:5000. 

### 4.7. Yeast Two-Hybrid Assay (GAL4 Transcription Factor-Based Assay)

The pDEST22 (PREY) and pDEST32 (BAIT) vectors (ProQuest Two-Hybrid System, Thermo Fisher scientific, Waltham, MA, USA) were used for analysis. The *S. cerevisiae* strain MaV203 (Thermo Fisher scientific, Waltham, MA, USA) was transformed simultaneously with pDEST22 and pDEST32 vectors. To generate *S. cerevisiae* MaV203 transformants, the protocol for the chemical preparation of cells was used [58]. As controls, a few pairs of vectors (pEXP32/Krev1 and pEXP22/RalGDS-wild type, pEXP22/RalGDS-m1 and pEXP22/RalGDS-m2) suggested by the manufacturer were used for strong, weak and not detectable interactions.

### 4.8. Histochemical Staining and Microscopy

Root samples were collected 14 days after plant inoculation and tested for deformations and curling of root hairs. Plants were removed from the pots and gently washed from vermiculite under running water at room temperature. Five plants per variant were used. Five to six lateral roots attached to the part of the main root located 2–3 cm from the cotyledons were taken from each plant. Segments of 4–8 cm in length from the tip of the root were stained with 0.01% methylene blue and used for analysis (at least 40 visual fields for each sample using 20× lens; about 350–400 visual fields in total). To analyze infection thread growth, 5–6 upper lateral roots from each plant were taken, washed three times for 5 min in water, then fixed with 1.25% glutaraldehyde buffered with 0.1 M PBS buffer (pH 7.2) for 15 min under vacuum (−0.9 bar; Vacuubrand ME 1C vacuum pump, Vacuubrand, Germany) followed by 1 h at atmospheric pressure and then rinsed with the PBS buffer 3 times for 10 min. Then, roots were stained with X-Gal overnight at room temperature as described [59]. After incubation, roots were washed with PBS and with distilled water (3 times for 10 min each). Before analysis, roots were cleared for 1 min in a 12% solution of commercial bleach, then washed 3 times in water. Pictures were taken using an Olympus BX51 microscope (Olympus Optical Co. Europa GmbH, Hamburg, Germany) equipped with a ColorView II digital camera and analySIS FIVE analytical software (Olympus Soft Imaging Solution GmbH, Hamburg, Germany). 

### 4.9. Analysis of Mycorrhizal Colonization

*Rhizophagus irregularis* (BEG144, International Bank of *Glomeromycota* (Dijon, France)) was kindly provided by Professor Dirk Redecker as a soil–root-based inoculum from onion (*Allium cepa* L.) pot cultures. To obtain mycorrhizal plants, pea seedlings were placed in a nurse pot system with chives (*Allium schoenoprasum* L.) as nurse plants and grown under conditions of a growth chamber as previously described [60]. A mineral substrate, silica-rich marl, was used as a growth substrate.

Mycorrhizal colonization was determined according to [61]. Lateral root samples were collected individually from each plant and stained with Sheaffer black ink [62]. Three parameters were considered: *M*%, the intensity of the internal colonization of the root system (reflects the proportion of the root length colonized by the fungus); *a*%, the arbuscule abundance in mycorrhizal root fragments (characterizes the functional state of the fungus); *v*%, the vesicle and/or spore abundance in mycorrhizal root fragments (reflects the developmental maturity of the fungus). One hundred and fifty random root pieces (1 cm) were scored per replicate (not less than 750 cm/genotype).

To localize fungal structures in root tissues, mycorrhizal roots were stained as described previously [63] with modifications. Roots were fixed in fixative solution (4% paraformaldehyde, 0.1% Tween-20, 0.1% Triton X-100) in 1/3 MTSB (50 mM PIPES, 5 mM MgSO4x7H2O, 5 mM EGTA, pH 6.9). Air from tissues was pumped out for 10 min at -0.9 bar three times, followed by overnight incubation at 4 °C. Then, roots were cleared in 20% KOH for 4 days at 4 °C, rinsed thoroughly with MTSB, and then incubated with 0.2 mkg/mL Alexa Fluor 488 WGA with 0.1% Tween-20, 0.1% Triton X-100 under fixation conditions. Then, roots were incubated in propidium iodide 10 mkg/mL to stain the cell wall. Stained roots were examined microscopically using a confocal microscope. 

### 4.10. Fusarium Culmorum Infection

Highly aggressive *Fusarium culmorum* (Wm. G. Sm.) Sacc. strain 334 was used for plant treatment [12]. To obtain inoculum, *F. culmorum* strains were grown on Chapek’s agar for 14 days and then washed from the plates with sterile water. Four-to-five-day old pea seedlings were transferred in pots with vermiculite saturated with Jensen medium [56], containing 10^5^ conidia of the fungus. The control plants were grown in sterile vermiculite saturated with Jensen medium. The assessment of the intensity of plant infection was carried out with a 5-point system (Table S5). 

### 4.11. Statistical Methods and Computer Software

An ANOVA (two-way ANOVA) was performed with R software version 3.5.1 (http://www.r-project.org/) for the two independent variables (plant type and bacterial strain), taking into account their interaction. To analyze the number of curled and deformed root hairs as well as infection threads, the size of each group in each repeat was 9–10 roots from different plants. Before analysis, the data were normalized to 100 fields of view and checked for normal distribution using the Shapiro–Wilk test. Pairwise comparisons were made using the Tukey test.

## 5. Conclusions

The analysis of structural variations in LysM domains of LysM-RLK *Ps*K1 showed different effects on pea–rhizobial symbiosis development. Experiments with rhizobial mutants indicated that *Ps*K1 function in infection thread growth and nodule development may depend on the Nod factor structure. It was shown that the amino acid P169 in LysM3 motif is important for *Ps*K1 and *Ps*SYM10 complex formation and subsequent signal transduction.

## Figures and Tables

**Table 1:** Mutations in the *k1* gene and their effects in mutant plants [25]. ECD: extracellular domain.

Mutant Line	Mutation	DNA Position	Protein Position	Localization
885 (*k1-1*)	G → A	1445	G332D	Kinase domain
817 (*k1-2*)	C → T	571	P169S	LysM3 motif of ECD
2265 (*k1-3*)	C → T	242	S59F	LysM1 motif of ECD

**Table 2:** Root-hair deformations and curling, infection thread (IT) growth in wild-type Cameor and *k1-2* mutant lines during inoculation with *R. leguminosarum* strains.

Variant	Root-Hair Deformations and Curling			
	A34 (wild type)	A42 (*nodE*)	A67 (*nodO*)	A91 (*nodE nodO*)
Cameor (wild type)	292.2 ± 2.6 ^b^	426.6 ± 3.5 ^a^	297.65 ± 4.9 ^b^	124.94 ± 3.3 ^d^
*k1-1* mutant	4.49 ± 0.2 ^f^	5.84 ± 0.27 ^f^	0	0
*k1-2* mutant	122.7 ± 2.7 ^d^	188.7 ± 3.3 ^c^	37.03 ± 1.33 ^e^	30.81 ± 0.7 * ^e^
*k1-3* mutant	297.2 ± 2.6 ^b^	434.1 ± 5.8 ^a^	294.9 ± 1.5 ^b^	120.2 ± 2 ^d^
	**Infection Thread Growth (number of ITs Aborted in Epidermis and Outer Cortex is Enclosed in Brackets)**			
	A34 (wild type)	A42 (*nodE*)	A67 (*nodO*)	A91 (*nodE nodO*)
Cameor (wild type)	22.9 ± 0.9 ^fg^	25.6 ± 0.9 ^f^	16.7 ± 0.5 ^g^	1028.99 ± 6.09 ^a^ (1020.82 ± 8.89)***
*k1-1* mutant**	2.85 ± 0.2 ^h^	4.28 ± 0.4 ^h^	0	0
*k1-2* mutant	141.2 ± 2.1 ^b^	139.1± 2.65 ^b^	33.3 ± 1.6 ^d^	0
	(99.6 ± 1.51)	(103.2±1.5)	(31.65 ± 1.4)	
*k1-3* mutant	43.9 ± 1.25 ^ce^	46.54± 1.1 ^c^	34.7 ± 1.6 ^de^	1035.6 ± 3.1 ^a^ (1029.8 ± 2.8)***

* Only root hair deformations, branching, curling, and the formation of microcolonies were observed. ** For *k1-1* line, only short aborted ITs were found in small root hairs. *** Most of the ITs were blocked in the epidermis; data represent the number of deformed and curled root hairs (± SEM) as well as ITs (± SEM) at 14 dai in 100 visual fields of the microscope. The size of each group in each repeat was 9–10 roots from different plants. Before analysis, a log transformation of the data was performed to conform to normality in order to calculate appropriate statistic values. For the better visual interpretation the data shown in the table, they are presented as the sum of counts normalized to 100 fields of view. Pairwise comparisons were made using Tukey’s test and the CLD algorithm was performed to summarize the sets of groups that differed significantly. Two-way ANOVA showed significant differences in the number of curled and deformed root hairs, depending on the bacterial strain (F(df = 3) = 593.12, *p* < 0.001) and depending on the type of plant (F(df = 3) = 3338.70, *p* < 0.001). The interaction of the two factors also turned out to be significant (F(df = 9) = 92.91, *p* < 0.001). Similar results were obtained by analyzing the number of infection threads depending on the bacterial strain (F(df = 3) = 4156.63, *p* < 0.001) and the type of plant (F(df = 3) = 3985.77, *p* < 0.001); the interaction of two factors in this the case had a more pronounced effect (F(df = 9) = 3054.87, *p* < 0.001). Values with different letters are significantly different (*p* < 0.01) as analyzed by two-way ANOVA and the Tukey test (Table S1 for root hair deformations and curling and Table S2 for infection thread growth).

## Supplementary Materials

Supplementary materials can be found at https://www.mdpi.com/1422-0067/20/7/1624/s1.
