# Supplementary material for: Structural Variations in LysM Domains of LysM-RLK PsK1 May Result in a Different Effect on Pea–Rhizobial Symbiosis Development
Source: Int J Mol Sci. 2019 Apr 1;20(7):1624. doi: 10.3390/ijms20071624 (PMC6479807; doi:10.3390/ijms20071624)

Table S1. Statistical analysis for root hair deformation and curling during inoculation of wild type (wt) plants, *k1-1* (mut885), *k1-2* (mut817) and *k1-3* (mut2265) mutant lines with different rhizobial strains A34 (wild type), A42 (*nodE*), A67 (*nodO*), A91 (*nodE nodO*) using Tukey test

	diff	p.adj
mut817:A34-mut2265:A34	-0.582268144	1.15E-14
mut885:A34-mut2265:A34	-1.335489542	1.15E-14
wt:A34-mut2265:A34	-0.011993735	1
mut2265:A42-mut2265:A34	0.295064745	1.60E-11
mut817:A42-mut2265:A34	-0.321364485	2.09E-13
mut885:A42-mut2265:A34	-1.32266738	1.15E-14
wt:A42-mut2265:A34	0.281241439	7.06E-12
mut2265:A67-mut2265:A34	-0.005228449	1
mut817:A67-mut2265:A34	-1.06695281	1.15E-14
mut885:A67-mut2265:A34	-1.379398224	1.15E-14
wt:A67-mut2265:A34	-0.003279643	1
mut2265:A91-mut2265:A34	-0.591435055	1.15E-14
mut817:A91-mut2265:A34	-1.10602768	1.15E-14
mut885:A91-mut2265:A34	-1.379398224	1.15E-14
wt:A91-mut2265:A34	-0.577136786	1.15E-14
mut885:A34-mut817:A34	-0.753221397	1.15E-14
wt:A34-mut817:A34	0.570274409	1.15E-14
mut2265:A42-mut817:A34	0.877332889	1.15E-14
mut817:A42-mut817:A34	0.26090366	1.12E-10
mut885:A42-mut817:A34	-0.740399235	1.15E-14
wt:A42-mut817:A34	0.863509584	1.15E-14
mut2265:A67-mut817:A34	0.577039695	1.15E-14
mut817:A67-mut817:A34	-0.484684665	1.17E-14
mut885:A67-mut817:A34	-0.79713008	1.15E-14
wt:A67-mut817:A34	0.578988501	1.15E-14
mut2265:A91-mut817:A34	-0.009166911	1
mut817:A91-mut817:A34	-0.523759535	1.15E-14
mut885:A91-mut817:A34	-0.79713008	1.15E-14
wt:A91-mut817:A34	0.005131358	1
wt:A34-mut885:A34	1.323495806	1.15E-14
mut2265:A42-mut885:A34	1.630554286	1.15E-14
mut817:A42-mut885:A34	1.014125057	1.15E-14
mut885:A42-mut885:A34	0.012822162	0.999999999
wt:A42-mut885:A34	1.616730981	1.15E-14
mut2265:A67-mut885:A34	1.330261093	1.15E-14
mut817:A67-mut885:A34	0.268536732	3.34E-11
mut885:A67-mut885:A34	-0.043908683	0.979548711
wt:A67-mut885:A34	1.332209898	1.15E-14
mut2265:A91-mut885:A34	0.744054487	1.15E-14
mut817:A91-mut885:A34	0.229461862	4.73E-09
mut885:A91-mut885:A34	-0.043908683	0.976260023
wt:A91-mut885:A34	0.758352755	1.15E-14

mut2265:A42-wt:A34	0.30705848	6.33E-13
mut817:A42-wt:A34	-0.309370749	2.08E-13
mut885:A42-wt:A34	-1.310673644	1.15E-14
wt:A42-wt:A34	0.293235175	2.97E-13
mut2265:A67-wt:A34	0.006765286	1
mut817:A67-wt:A34	-1.054959074	1.15E-14
mut885:A67-wt:A34	-1.367404489	1.15E-14
wt:A67-wt:A34	0.008714092	1
mut2265:A91-wt:A34	-0.579441319	1.15E-14
mut817:A91-wt:A34	-1.094033944	1.15E-14
mut885:A91-wt:A34	-1.367404489	1.15E-14
wt:A91-wt:A34	-0.565143051	1.15E-14
mut817:A42-mut2265:A42	-0.616429229	1.15E-14
mut885:A42-mut2265:A42	-1.617732124	1.15E-14
wt:A42-mut2265:A42	-0.013823305	0.999999998
mut2265:A67-mut2265:A42	-0.300293193	7.45E-12
mut817:A67-mut2265:A42	-1.362017554	1.15E-14
mut885:A67-mut2265:A42	-1.674462969	1.15E-14
wt:A67-mut2265:A42	-0.298344388	6.12E-13
mut2265:A91-mut2265:A42	-0.886499799	1.15E-14
mut817:A91-mut2265:A42	-1.401092424	1.15E-14
mut885:A91-mut2265:A42	-1.674462969	1.15E-14
wt:A91-mut2265:A42	-0.872201531	1.15E-14
mut885:A42-mut817:A42	-1.001302895	1.15E-14
wt:A42-mut817:A42	0.602605924	1.15E-14
mut2265:A67-mut817:A42	0.316136036	2.80E-13
mut817:A67-mut817:A42	-0.745588325	1.15E-14
mut885:A67-mut817:A42	-1.05803374	1.15E-14
wt:A67-mut817:A42	0.318084842	1.50E-13
mut2265:A91-mut817:A42	-0.27007057	6.47E-12
mut817:A91-mut817:A42	-0.784663195	1.15E-14
mut885:A91-mut817:A42	-1.05803374	1.15E-14
wt:A91-mut817:A42	-0.255772301	2.16E-11
wt:A42-mut885:A42	1.603908819	1.15E-14
mut2265:A67-mut885:A42	1.317438931	1.15E-14
mut817:A67-mut885:A42	0.25571457	2.54E-10
mut885:A67-mut885:A42	-0.056730845	0.848314805
wt:A67-mut885:A42	1.319387736	1.15E-14
mut2265:A91-mut885:A42	0.731232325	1.15E-14
mut817:A91-mut885:A42	0.2166397	3.58E-08
mut885:A91-mut885:A42	-0.056730845	0.831683693
wt:A91-mut885:A42	0.745530594	1.15E-14
mut2265:A67-wt:A42	-0.286469888	3.17E-12
mut817:A67-wt:A42	-1.348194249	1.15E-14
mut885:A67-wt:A42	-1.660639664	1.15E-14
wt:A67-wt:A42	-0.284521083	2.70E-13
mut2265:A91-wt:A42	-0.872676494	1.15E-14

mut817:A91-wt:A42	-1.387269119	1.15E-14
mut885:A91-wt:A42	-1.660639664	1.15E-14
wt:A91-wt:A42	-0.858378225	1.15E-14
mut817:A67-mut2265:A67	-1.061724361	1.15E-14
mut885:A67-mut2265:A67	-1.374169775	1.15E-14
wt:A67-mut2265:A67	0.001948806	1
mut2265:A91-mut2265:A67	-0.586206606	1.15E-14
mut817:A91-mut2265:A67	-1.100799231	1.15E-14
mut885:A91-mut2265:A67	-1.374169775	1.15E-14
wt:A91-mut2265:A67	-0.571908337	1.15E-14
mut885:A67-mut817:A67	-0.312445415	1.20E-13
wt:A67-mut817:A67	1.063673166	1.15E-14
mut2265:A91-mut817:A67	0.475517755	1.15E-14
mut817:A91-mut817:A67	-0.03907487	0.996735164
mut885:A91-mut817:A67	-0.312445415	1.05E-13
wt:A91-mut817:A67	0.489816024	1.15E-14
wt:A67-mut885:A67	1.376118581	1.15E-14
mut2265:A91-mut885:A67	0.78796317	1.15E-14
mut817:A91-mut885:A67	0.273370545	1.85E-13
mut885:A91-mut885:A67	-4.16E-17	1
wt:A91-mut885:A67	0.802261438	1.15E-14
mut2265:A91-wt:A67	-0.588155412	1.15E-14
mut817:A91-wt:A67	-1.102748037	1.15E-14
mut885:A91-wt:A67	-1.376118581	1.15E-14
wt:A91-wt:A67	-0.573857143	1.15E-14
mut817:A91-mut2265:A91	-0.514592625	1.15E-14
mut885:A91-mut2265:A91	-0.78796317	1.15E-14
wt:A91-mut2265:A91	0.014298269	0.999999983
mut885:A91-mut817:A91	-0.273370545	1.59E-13
wt:A91-mut817:A91	0.528890894	1.15E-14
wt:A91-mut885:A91	0.802261438	1.15E-14

Table S2. Statistical analysis for infection threads development during inoculation wild type (wt) plants, *k1-1* (mut885), *k1-2* (mut817) and *k1-3* (mut2265) mutant lines with different rhizobial strains strains A34 (wild type), A42 (*nodE*), A67 (*nodO*), A91 (*nodE nodO*) using Tukey test

	diff	p.adj
mut817:A34-mut2265:A34	0.515411097	1.15E-14
mut885:A34-mut2265:A34	-0.335984586	1.15E-14
wt:A34-mut2265:A34	-0.156657299	8.29E-09
mut2265:A42-mut2265:A34	0.018038649	0.999984183
mut817:A42-mut2265:A34	0.506274655	1.15E-14
mut885:A42-mut2265:A34	-0.322753872	1.17E-14
wt:A42-mut2265:A34	-0.136779837	2.86E-07
mut2265:A67-mut2265:A34	-0.06826263	0.170646369
mut817:A67-mut2265:A34	-0.080025537	0.028312176
mut885:A67-mut2265:A34	-0.36197377	1.15E-14
wt:A67-mut2265:A34	-0.209497884	1.59E-13
mut2265:A91-mut2265:A34	2.066136918	1.15E-14
mut817:A91-mut2265:A34	-0.364218553	1.15E-14
mut885:A91-mut2265:A34	-0.364218553	1.15E-14
wt:A91-mut2265:A34	2.059206671	1.15E-14
mut885:A34-mut817:A34	-0.851395683	1.15E-14
wt:A34-mut817:A34	-0.672068397	1.15E-14
mut2265:A42-mut817:A34	-0.497372448	1.15E-14
mut817:A42-mut817:A34	-0.009136442	0.999999996
mut885:A42-mut817:A34	-0.838164969	1.15E-14
wt:A42-mut817:A34	-0.652190934	1.15E-14
mut2265:A67-mut817:A34	-0.583673727	1.15E-14
mut817:A67-mut817:A34	-0.595436634	1.15E-14
mut885:A67-mut817:A34	-0.877384868	1.15E-14
wt:A67-mut817:A34	-0.724908981	1.15E-14
mut2265:A91-mut817:A34	1.55072582	1.15E-14
mut817:A91-mut817:A34	-0.879629651	1.15E-14
mut885:A91-mut817:A34	-0.879629651	1.15E-14
wt:A91-mut817:A34	1.543795574	1.15E-14
wt:A34-mut885:A34	0.179327286	7.46E-12
mut2265:A42-mut885:A34	0.354023235	1.15E-14
mut817:A42-mut885:A34	0.842259241	1.15E-14
mut885:A42-mut885:A34	0.013230713	0.999999307
wt:A42-mut885:A34	0.199204749	1.66E-13
mut2265:A67-mut885:A34	0.267721956	2.23E-14
mut817:A67-mut885:A34	0.255959049	2.35E-14
mut885:A67-mut885:A34	-0.025989185	0.990978758
wt:A67-mut885:A34	0.126486702	8.38E-07
mut2265:A91-mut885:A34	2.402121503	1.15E-14
mut817:A91-mut885:A34	-0.028233968	0.989567703
mut885:A91-mut885:A34	-0.028233968	0.976918986
wt:A91-mut885:A34	2.395191256	1.15E-14

mut2265:A42-wt:A34	0.174695949	1.25E-10
mut817:A42-wt:A34	0.662931955	1.15E-14
mut885:A42-wt:A34	-0.166096573	1.92E-10
wt:A42-wt:A34	0.019877462	0.999785297
mut2265:A67-wt:A34	0.08839467	0.007941224
mut817:A67-wt:A34	0.076631763	0.029404436
mut885:A67-wt:A34	-0.205316471	1.03E-13
wt:A67-wt:A34	-0.052840585	0.393226411
mut2265:A91-wt:A34	2.222794217	1.15E-14
mut817:A91-wt:A34	-0.207561254	1.43E-13
mut885:A91-wt:A34	-0.207561254	8.29E-14
wt:A91-wt:A34	2.21586397	1.15E-14
mut817:A42-mut2265:A42	0.488236006	1.15E-14
mut885:A42-mut2265:A42	-0.340792521	1.15E-14
wt:A42-mut2265:A42	-0.154818486	4.33E-09
mut2265:A67-mut2265:A42	-0.086301279	0.017873892
mut817:A67-mut2265:A42	-0.098064186	0.001560807
mut885:A67-mut2265:A42	-0.38001242	1.15E-14
wt:A67-mut2265:A42	-0.227536533	1.13E-13
mut2265:A91-mut2265:A42	2.048098268	1.15E-14
mut817:A91-mut2265:A42	-0.382257203	1.15E-14
mut885:A91-mut2265:A42	-0.382257203	1.15E-14
wt:A91-mut2265:A42	2.041168022	1.15E-14
mut885:A42-mut817:A42	-0.829028527	1.15E-14
wt:A42-mut817:A42	-0.643054492	1.15E-14
mut2265:A67-mut817:A42	-0.574537285	1.15E-14
mut817:A67-mut817:A42	-0.586300192	1.15E-14
mut885:A67-mut817:A42	-0.868248426	1.15E-14
wt:A67-mut817:A42	-0.715772539	1.15E-14
mut2265:A91-mut817:A42	1.559862262	1.15E-14
mut817:A91-mut817:A42	-0.870493209	1.15E-14
mut885:A91-mut817:A42	-0.870493209	1.15E-14
wt:A91-mut817:A42	1.552932016	1.15E-14
wt:A42-mut885:A42	0.185974035	4.62E-13
mut2265:A67-mut885:A42	0.254491242	4.63E-14
mut817:A67-mut885:A42	0.242728336	5.17E-14
mut885:A67-mut885:A42	-0.039219898	0.770015761
wt:A67-mut885:A42	0.113255988	1.68E-05
mut2265:A91-mut885:A42	2.38889079	1.15E-14
mut817:A91-mut885:A42	-0.041464681	0.785630338
mut885:A91-mut885:A42	-0.041464681	0.666351204
wt:A91-mut885:A42	2.381960543	1.15E-14
mut2265:A67-wt:A42	0.068517207	0.098741244
mut817:A67-wt:A42	0.0567543	0.274642219
mut885:A67-wt:A42	-0.225193933	2.20E-14
wt:A67-wt:A42	-0.072718047	0.024909088
mut2265:A91-wt:A42	2.202916755	1.15E-14

mut817:A91-wt:A42	-0.227438716	4.80E-14
mut885:A91-wt:A42	-0.227438716	1.62E-14
wt:A91-wt:A42	2.195986508	1.15E-14
mut817:A67-mut2265:A67	-0.011762907	0.999999923
mut885:A67-mut2265:A67	-0.293711141	1.17E-14
wt:A67-mut2265:A67	-0.141235254	1.03E-07
mut2265:A91-mut2265:A67	2.134399547	1.15E-14
mut817:A91-mut2265:A67	-0.295955924	1.18E-14
mut885:A91-mut2265:A67	-0.295955924	1.15E-14
wt:A91-mut2265:A67	2.127469301	1.15E-14
mut885:A67-mut817:A67	-0.281948234	1.17E-14
wt:A67-mut817:A67	-0.129472347	4.17E-07
mut2265:A91-mut817:A67	2.146162454	1.15E-14
mut817:A91-mut817:A67	-0.284193017	1.18E-14
mut885:A91-mut817:A67	-0.284193017	1.15E-14
wt:A91-mut817:A67	2.139232207	1.15E-14
wt:A67-mut885:A67	0.152475886	1.95E-11
mut2265:A91-mut885:A67	2.428110688	1.15E-14
mut817:A91-mut885:A67	-0.002244783	1
mut885:A91-mut885:A67	-0.002244783	1
wt:A91-mut885:A67	2.421180441	1.15E-14
mut2265:A91-wt:A67	2.275634802	1.15E-14
mut817:A91-wt:A67	-0.154720669	2.27E-10
mut885:A91-wt:A67	-0.154720669	4.75E-12
wt:A91-wt:A67	2.268704555	1.15E-14
mut817:A91-mut2265:A91	-2.430355471	1.15E-14
mut885:A91-mut2265:A91	-2.430355471	1.15E-14
wt:A91-mut2265:A91	-0.006930247	1
mut885:A91-mut817:A91	-4.44E-16	1
wt:A91-mut817:A91	2.423425224	1.15E-14
wt:A91-mut885:A91	2.423425224	1.15E-14

Table S3. Statistical analysis for nodules during inoculation wild type (wt) plants, *k1-1* (mut885), *k1-2* (mut817) and *k1-3* (mut2265) mutant lines with different rhizobial strains A34 (wild type), A42 (*nodE*), A67 (*nodO*), A91 (*nodE nodO*) using Tukey test

	diff	p.adj
mut817:A34-mut2265:A34	-3.71297658	0
wt:A34-mut2265:A34	0.093625139	0.498509995
mut2265:A42-mut2265:A34	-0.313789266	3.96E-08
mut817:A42-mut2265:A34	-3.71297658	0
wt:A42-mut2265:A34	-0.174250378	0.004772343
mut2265:A67-mut2265:A34	0.013866939	0.99999994
mut817:A67-mut2265:A34	-3.71297658	0
wt:A67-mut2265:A34	0.028098648	0.999910064
mut2265:A91-mut2265:A34	-1.021529714	0
mut817:A91-mut2265:A34	-3.71297658	0
wt:A91-mut2265:A34	-0.967876916	0
wt:A34-mut817:A34	3.806601719	0
mut2265:A42-mut817:A34	3.399187314	0
mut817:A42-mut817:A34	1.94E-16	1
wt:A42-mut817:A34	3.538726202	0
mut2265:A67-mut817:A34	3.726843519	0
mut817:A67-mut817:A34	1.67E-16	1
wt:A67-mut817:A34	3.741075228	0
mut2265:A91-mut817:A34	2.691446867	0
mut817:A91-mut817:A34	3.89E-16	1
wt:A91-mut817:A34	2.745099664	0
mut2265:A42-wt:A34	-0.407414405	1.14E-11
mut817:A42-wt:A34	-3.806601719	0
wt:A42-wt:A34	-0.267875517	2.10E-06
mut2265:A67-wt:A34	-0.0797582	0.723628056
mut817:A67-wt:A34	-3.806601719	0
wt:A67-wt:A34	-0.065526491	0.90042761
mut2265:A91-wt:A34	-1.115154853	0
mut817:A91-wt:A34	-3.806601719	0
wt:A91-wt:A34	-1.061502055	0
mut817:A42-mut2265:A42	-3.399187314	0
wt:A42-mut2265:A42	0.139538888	0.052748114
mut2265:A67-mut2265:A42	0.327656204	1.20E-08
mut817:A67-mut2265:A42	-3.399187314	0
wt:A67-mut2265:A42	0.341887914	3.56E-09
mut2265:A91-mut2265:A42	-0.707740448	0
mut817:A91-mut2265:A42	-3.399187314	0
wt:A91-mut2265:A42	-0.654087651	0
wt:A42-mut817:A42	3.538726202	0
mut2265:A67-mut817:A42	3.726843519	0
mut817:A67-mut817:A42	-2.78E-17	1
wt:A67-mut817:A42	3.741075228	0

mut2265:A91-mut817:A42	2.691446867	0
mut817:A91-mut817:A42	1.94E-16	1
wt:A91-mut817:A42	2.745099664	0
mut2265:A67-wt:A42	0.188117317	0.001648452
mut817:A67-wt:A42	-3.538726202	0
wt:A67-wt:A42	0.202349026	0.0005306
mut2265:A91-wt:A42	-0.847279336	0
mut817:A91-wt:A42	-3.538726202	0
wt:A91-wt:A42	-0.793626538	0
mut817:A67-mut2265:A67	-3.726843519	0
wt:A67-mut2265:A67	0.014231709	0.999999921
mut2265:A91-mut2265:A67	-1.035396652	0
mut817:A91-mut2265:A67	-3.726843519	0
wt:A91-mut2265:A67	-0.981743855	0
wt:A67-mut817:A67	3.741075228	0
mut2265:A91-mut817:A67	2.691446867	0
mut817:A91-mut817:A67	2.22E-16	1
wt:A91-mut817:A67	2.745099664	0
mut2265:A91-wt:A67	-1.049628362	0
mut817:A91-wt:A67	-3.741075228	0
wt:A91-wt:A67	-0.995975564	0
mut817:A91-mut2265:A91	-2.691446867	0
wt:A91-mut2265:A91	0.053652797	0.973699575
wt:A91-mut817:A91	2.745099664	0

Table S4. Statistical analysis for primordium during inoculation wild type (wt) plants, *k1-1* (mut885), *k1-2* (mut817) and *k1-3* (mut2265) mutant lines with different rhizobial strains A34 (wild type), A42 (*nodE*), A67 (*nodO*), A91 (*nodE nodO*) using Tukey test

	diff	p.adj
mut817:A34-mut2265:A34	-0.875821385	3.52E-11
wt:A34-mut2265:A34	-0.026253277	0.999999989
mut2265:A42-mut2265:A34	-0.131958317	0.944931209
mut817:A42-mut2265:A34	-0.973450149	0
wt:A42-mut2265:A34	-0.101128181	0.992410614
mut2265:A67-mut2265:A34	0.056142681	0.999967323
mut817:A67-mut2265:A34	-2.790901398	0
wt:A67-mut2265:A34	-0.009337423	1
mut2265:A91-mut2265:A34	-1.935568174	0
mut817:A91-mut2265:A34	-2.790901398	0
wt:A91-mut2265:A34	-1.715845716	0
wt:A34-mut817:A34	0.849568108	9.99E-11
mut2265:A42-mut817:A34	0.743863068	5.77E-09
mut817:A42-mut817:A34	-0.097628764	0.991879625
wt:A42-mut817:A34	0.774693204	1.76E-09
mut2265:A67-mut817:A34	0.931964066	1.17E-12
mut817:A67-mut817:A34	-1.915080013	0
wt:A67-mut817:A34	0.866483962	5.14E-11
mut2265:A91-mut817:A34	-1.059746789	0
mut817:A91-mut817:A34	-1.915080013	0
wt:A91-mut817:A34	-0.840024332	1.44E-10
mut2265:A42-wt:A34	-0.10570504	0.989152769
mut817:A42-wt:A34	-0.947196872	0
wt:A42-wt:A34	-0.074874904	0.999462799
mut2265:A67-wt:A34	0.082395958	0.998704409
mut817:A67-wt:A34	-2.764648121	0
wt:A67-wt:A34	0.016915854	1
mut2265:A91-wt:A34	-1.909314897	0
mut817:A91-wt:A34	-2.764648121	0
wt:A91-wt:A34	-1.689592439	0
mut817:A42-mut2265:A42	-0.841491831	3.10E-11
wt:A42-mut2265:A42	0.030830136	0.999999937
mut2265:A67-mut2265:A42	0.188100998	0.640197365
mut817:A67-mut2265:A42	-2.658943081	0
wt:A67-mut2265:A42	0.122620895	0.966746573
mut2265:A91-mut2265:A42	-1.803609857	0
mut817:A91-mut2265:A42	-2.658943081	0
wt:A91-mut2265:A42	-1.583887399	0

wt:A42-mut817:A42	0.872321967	6.85E-12
mut2265:A67-mut817:A42	1.029592829	0
mut817:A67-mut817:A42	-1.817451249	0
wt:A67-mut817:A42	0.964112726	0
mut2265:A91-mut817:A42	-0.962118025	0
mut817:A91-mut817:A42	-1.817451249	0
wt:A91-mut817:A42	-0.742395568	1.71E-09
mut2265:A67-wt:A42	0.157270862	0.84218217
mut817:A67-wt:A42	-2.689773217	0
wt:A67-wt:A42	0.091790759	0.996637571
mut2265:A91-wt:A42	-1.834439993	0
mut817:A91-wt:A42	-2.689773217	0
wt:A91-wt:A42	-1.614717535	0
mut817:A67-mut2265:A67	-2.847044079	0
wt:A67-mut2265:A67	-0.065480103	0.999850487
mut2265:A91-mut2265:A67	-1.991710855	0
mut817:A91-mut2265:A67	-2.847044079	0
wt:A91-mut2265:A67	-1.771988397	0
wt:A67-mut817:A67	2.781563975	0
mut2265:A91-mut817:A67	0.855333224	7.98E-11
mut817:A91-mut817:A67	-2.22E-16	1
wt:A91-mut817:A67	1.075055682	0
mut2265:A91-wt:A67	-1.926230751	0
mut817:A91-wt:A67	-2.781563975	0
wt:A91-wt:A67	-1.706508294	0
mut817:A91-mut2265:A91	-0.855333224	7.98E-11
wt:A91-mut2265:A91	0.219722458	0.408517066
wt:A91-mut817:A91	1.075055682	0

Table S5. The visual disease symptoms caused by *Fusarium culmorum* in pea roots (in points)

Points	Visual disease symptoms
0	The healthy plant with no signs of infection.
1	There are signs of “banding” on the roots, death of individual root cells, without obvious disease symptoms.
2	Numerous black stripes and spots on the roots. The tips of the main and lateral roots are necrotized, characteristic thickening and deformation of the roots are noticeable.
3	Plant necrosis zones are well visible on the main and lateral roots. The development of main root is blocked.
4	Signs of root infection (necrosis) are strongly pronounced, the plant is severely inhibited. The death of plants is observed.
5	Complete death of the plant

Table S6. List of primers

Name	Sequence
Primers for *K1* mutagenesis	
*K1_k1_2*_mut_F	5'-GTTTGTTACTTATTCGTTAAGGTCTACGG-3'
*K1_k1_2*_mut_R	5'-AAGCCATAATCTTTTGAAATCTGGCT-3'
*K1_k1_3*_mut_F	5'-TAGTTACCAACTTTTCTGATGTTTT-3'
*K1_k1_3*_mut_R	5'-TCTTTGATTGCATATAGTTTT-3'
Primers for qRT-PCR	
*PR10*_F	5'-GCCGGAACCATCAAGAAACT-3'
*PR10*_R	5'-GCCTTGAAAAGACCATCACCC-3'
*PAL2*_F	5'-GAGAATCAACACACTTCTCCAAGG-3'
*PAL2*_R	5'-GCATTAAGTATTTCTCCAGACGGTC-3'
*PUB22*_F	5'-TGTCCGATCACAAAACAACAGC-3'
*PUB22*_R	5'-GAAGCTTCTTTGATGAGTTTCGC-3'
*WRKY33*_F	5'-ACCCGAATTGTCCACGAAGAA-3'
*WRKY33*_R	5'-TTAGCAGGAACAATCACAAGGCA-3'
*PT4*_F	5'-CTTCACGTGCCATGTTCATC-3'
*PT4*_R	5'-GCGTCGGAAACAGCTCC-3'
*TI*_F	5'-ACCTTACAGCGTGAGCCTATAAGA-3'
*TI*_R	5'-GCGGCCGAGGTACGAAAGGTG-3'
*DELLA3*_F	5'-CGGATGAGCGGGACAACC-3'
*DELLA3*_R	5'-GCAATATAGAATTAACCGCCACAAC-3'
*Actin*_F	5'-CTCAGCACCTTCCAGCAGATGTG-3'
Actin _R	5'-CTTCTTATCCATGGCAACATAGTTC-3'
Ubiquitin _F	5'-TGAGAATCAACACACTTCTCCAAGG-3'
Ubiquitin _R	5'-GCATTAAGAATTTCCCCAGAGGTC-3'

Figure S1. View of wild type, *k1-1* and *k1-2* mutant roots with disease symptoms (a) and root and stem length (b) upon *Fusarium culmorum* infection on 8 and 16 days. The data of three independent biological experiments were analyzed. Values with different letters indicate significant differences compared to untreated plants based on Student's t-test ($P < 0.05$).

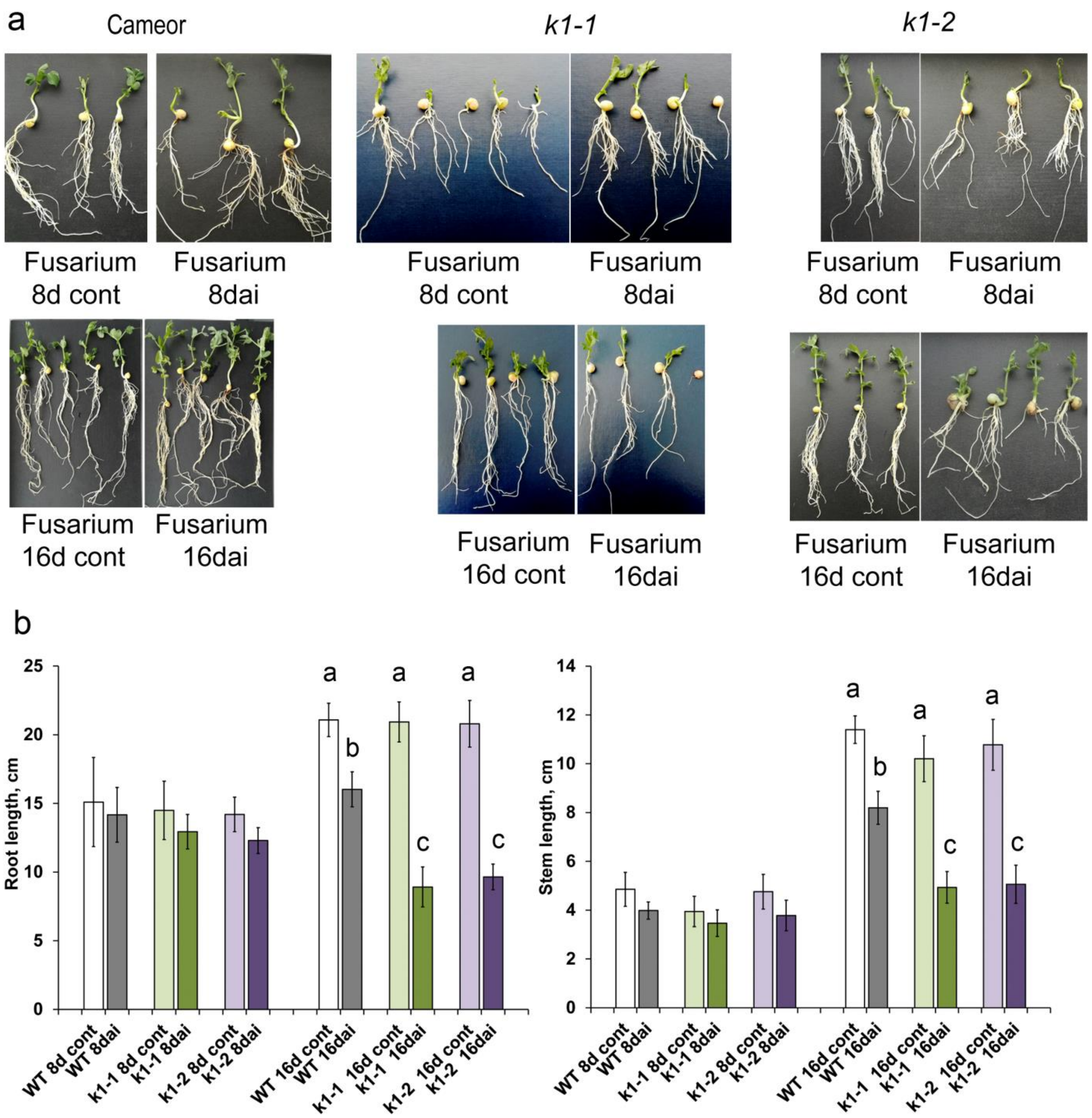

Supplement: Supplementary file 1 [file ijms-20-01624-s001.pdf]
